# In Vivo Effect of a Nisin–Biogel on the Antimicrobial and Virulence Signatures of Canine Oral *Enterococci*

**DOI:** 10.3390/antibiotics12030468

**Published:** 2023-02-25

**Authors:** Eva Cunha, Ana Filipa Ferreira, Sara Valente, Alice Matos, Luís Miguel Carreira, Marta Videira, Lélia Chambel, Luís Tavares, Manuela Oliveira

**Affiliations:** 1 CIISA—Centre for Interdisciplinary Research in Animal Health, Faculty of Veterinary Medicine, University of Lisbon, Av. da Universidade Técnica, 1300-477 Lisboa, Portugal; 2 Associate Laboratory for Animal and Veterinary Sciences (AL4AnimalS), 1300-477 Lisboa, Portugal; 3 Casa dos Animais de Lisboa, Estrada da Pimenteira,1300-459 Lisboa, Portugal; 4 BioISI—Biosystems and Integrative Sciences Institute, Faculdade de Ciências da Universidade de Lisboa, 1749-016 Lisboa, Portugal

**Keywords:** periodontal disease, dogs, nisin–biogel, *enterococci*, antimicrobial resistance, virulence signatures

## Abstract

Periodontal disease is a relevant oral disease in dogs and nisin–biogel has been previously proposed to be used in its control. *Enterococci*, as inhabitants of the oral cavity with a high genetic versatility, are a reliable bacterial model for antimicrobial studies. Our goal was to evaluate the in vivo influence of the long-term dental application of the nisin–biogel on the virulence and antimicrobial signatures of canine oral *enterococci*. Twenty dogs were randomly allocated to one of two groups (treatment group—TG with nisin–biogel dental application, or control group—CG without treatment) and submitted to dental plaque sampling at day 0 and after 90 days (T90). Samples were processed for *Enterococcus* spp. isolation, quantification, identification, molecular typing and antimicrobial and virulence characterization. From a total of 140 *enterococci*, molecular typing allowed us to obtain 70 representative isolates, mostly identified as *E. faecalis* and *E. faecium*. No significant differences (*p* > 0.05) were observed in the virulence index of the isolates obtained from samples collected from the TG and CG at T90. At T90, a statistically significant difference (*p* = 0.0008) was observed in the antimicrobial resistance index between the isolates from the TC and CG. Oral *enterococci* were revealed to be reservoirs of high resistant and virulent phenotypes.

## 1. Introduction

*Enterococci* are commensal inhabitants of the intestinal tract of humans and other mammals [1]. These bacteria have a high genome plasticity, resulting in the ability of acquiring, conserving and disseminating genetic determinants, being an interesting bacterial model for antimicrobial studies [2,3]. Nevertheless, *Enterococcus faecium*, classified by the World Health Organizaion (WHO) as a high-priority pathogen for the research and development of new antimicrobial compounds, along with other enterococcal species, may become an opportunistic pathogen and be associated with life-threatening infections [2,4,5]. If present in the dental plaque microbiota, *enterococci* can participate in chronic endodontic lesions and periodontitis, being associated with systemic consequences both in humans and dogs [6,7,8,9]. Among *Enterococcus* species, *E. faecium* and *E. faecalis* are the two most common species isolated from clinical specimens in dogs [9,10,11].

Periodontal disease (PD) is a widespread oral inflammatory disease, presenting high impact in the veterinary field. Studies describe PD prevalences higher than 80% in dogs over 2 years of age, reaching 100% in some breeds [12,13]. Additionally, PD may be associated with several local and systemic consequences, reinforcing the impact of this disease on global animal health [14,15,16,17]. Considering that, new therapeutic and preventative approaches are required to control PD in these animals. 

Previous studies have focused on the potential of the antimicrobial peptide nisin incorporated in a guar gum biogel as a promising compound for PD control in dogs [3,18,19,20]. Cunha and collaborators have demonstrated that the nisin–biogel has anti-biofilm activity against different bacterial species from the canine dental plaque, keeping its antimicrobial activity in the presence of canine saliva and over two years of storage at different temperatures [3,19,20]. This anti-biofilm ability may contribute to the prevention of biofilm formation by inhibiting the bacterial dental attachment, but the nisin–biogel can also act on mature biofilms, since nisin can penetrate the biofilm structure without being inactivated by its matrix, leading to bacterial death and biofilm destruction [3,19,21,22]. Additionally, the nisin–biogel showed no toxicity against eukaryotic cells [20]. However, previous in vitro studies have revealed that nisin may induce changes in the antimicrobial resistance profile of *enterococci* [23].

In order to understand the in vivo impact of the dental application of the nisin–biogel to dogs on the virulence and antimicrobial profile of oral *enterococci*, we evaluated the influence of the in vivo long-term dental application of the nisin–biogel in the virulence and antimicrobial signatures of oral *enterococci*, using samples collected during a previous randomized controlled clinical trial with dogs.

## 2. Results

Supragingival dental plaque samples were collected from twenty dogs at two timepoints (T0—timepoint 0, beginning of the study; T90—timepoint 90, 90 days after). Each animal was allocated to one of two groups: the control group (*n* = 10), to which no treatment was applied, or the treatment group (*n* = 10), in which each animal was submitted to a dental, topical application of the nisin–biogel (200 µg/mL), each 48 h. Then, samples were processed for *Enterococcus* spp. isolation, quantification, identification, molecular typing and antimicrobial and virulence characterization.

### 2.1. Enterococci Identification and Typing

At T0, 17 animals were positive for oral *enterococci*, while at T90, 18 animals were positive for this bacterial group. From each positive sample, four colonies with macroscopic morphology compatible with *enterococci* were selected, allowing us to collect a total of 68 isolates from samples obtained at T0 and 72 isolates from those collected at T90, in a total of 140 isolates. Mean *enterococci* counts obtained from T0 samples were of 3 × 10^7^ CFU/mL, while in T90 samples a reduction in bacterial quantification was observed, with a mean count of 5.8 × 10^5^ CFU/mL. Considering only the treatment group, T0 samples presented an *enterococci* concentration of 2.2 × 10^7^ CFU/mL, while in the T90 samples the concentration was 5.9 × 10^5^ CFU/mL. In the control group, a value of 4.1 × 10^7^ CFU/mL was obtained in the T0 samples, while in the T90 samples an *enterococci* count of 5.6 × 10^5^ CFU/mL was observed.

Isolate’s genotyping allowed us to gather a collection of 70 representative isolates, including 38 *enterococci* collected from the T0 samples and 32 isolates from the T90 samples (See Supplementary File S1).

**Figure 1:**
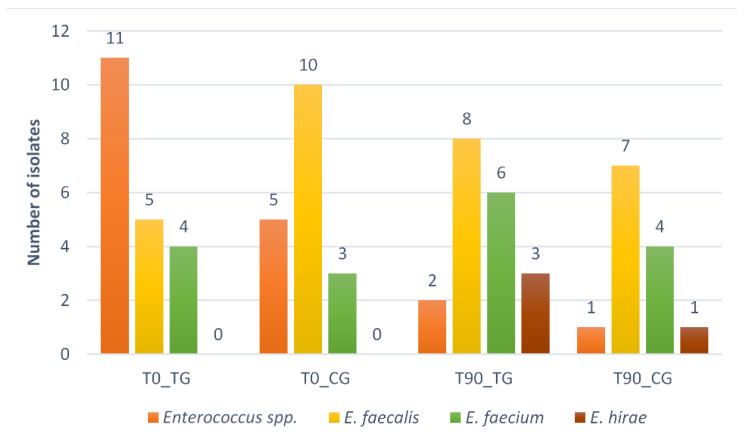
Species distribution by timepoint and by test group (control group and treatment group). T0—timepoint 0; T90—timepoint 90; TG—treatment group; CG—control group.

Species identification of the 38 *enterococci* recovered from the T0 samples revealed that 39.47% (*n* = 15/38) of the isolates belonged to the species *E. faecalis*, 18.42% (*n* = 7/38) to *E. faecium* and 42.11% (*n* = 16/38) were identified as *Enterococcus* spp. (Figure 1). Considering the isolates recovered from samples collected at the end of the clinical trial (timepoint 90), species distribution was as follows: 46.88% (*n* = 15/32) of the isolates were identified as *E. faecalis*, 31.25% (*n* = 10/32) as *E. faecium*, 12.50% (*n* = 4/32) as *E. hirae* and 9.34% (*n* = 3/32) as *Enterococcus* spp. (Figure 1). *E. durans* isolates were not detected in any of the oral samples.

### 2.2. Enterococci Virulence Signatures 

The number of isolates that phenotypically expressed the virulence determinants under study is described in Table 1, being organized by timepoint and animal group. The ability of producing biofilm was the most prevalent virulence factor detected in this study, regardless of the sample group or timepoint, followed by lipase and haemolysin production. 

The isolates mean virulence index is presented in Table 1. A slight increase in the mean virulence index was observed in the isolates from the TG samples collected at T0 and T90, but without statistical significance (*p*-value > 0.05). No significant differences (*p*-value > 0.05) were observed when comparing the virulence index of the isolates obtained from the TG and CG samples collected after nisin–biogel application at T90.

### 2.3. Enterococci Antimicrobial Resistance Profile

It was possible to observe a mean multiple antimicrobial resistance (MAR) index equal or higher than 0.4 in all the isolates from the bacterial collection, independently of the timepoint or test group of origin.

At the end of the clinical trial (T90), a statistically significant difference (*p* = 0.0008) was observed between the mean MAR index of the isolates from the treatment and control groups. Additionally, the MAR values increased from timepoint 0 to timepoint 90 in both groups (TG and CG) (Table 2).

Isolates were classified as showing multidrug resistance (MDR) when they were non-susceptible to at least one agent in three or more different antimicrobial classes [24]. A high MDR prevalence was detected in the isolates under study, independently of the timepoint or test group, ranging from 90 to 100% (Table 2). Considering high-level aminoglycoside resistance (HLAR) determination, the control group showed a higher number of positive isolates in comparison to the treatment group (Table 2).

## 3. Discussion

Antimicrobial peptides (AMPs) are relevant molecules in the fight against antimicrobial resistance dissemination. Until now, several AMPs have been investigated with this aim, with nisin being one of the most well-studied compounds from this antimicrobial class [25]. Recent reports have shown promising results from in vitro and in vivo studies regarding nisin and nisin–biogel use for canine PD control [3,18,19,20,26]. PD onset includes the formation of a dental plaque polymicrobial biofilm composed by several bacterial species [27,28,29,30]. Among this complex biofilm, *enterococci* can be found, being commensal inhabitants of the oral cavity of dogs, with high genetic plasticity [8].

This study analysed oral samples obtained from animals that participated in a previous clinical trial [31] in which the efficacy of the administration of the nisin–biogel for PD prevention in dogs was evaluated. In the present work, *enterococci* were used as bacterial models to study the in vivo influence of nisin–biogel on virulence and antimicrobial resistance profiles. It was possible to isolate *enterococci* from the samples collected from the majority of dogs at the start (*n* = 17/20) and at the end (*n* = 18/20) of the clinical study, allowing us to observe a reduction in *enterococci* counts from T0 to T90 in the animals from both test groups (CG or TG). Considering that, we believe that the dental application of the nisin–biogel had no direct effect on bacterial counts, and the observed reduction may be attributed to the dental plaque scaling procedure performed on all animals at T0, as suggested by other studies [32,33,34]. In fact, the animals from both groups (CG and TC) showed a reduction in their periodontal indices (gingivitis, dental plaque accumulation and periodontal pocket depth) at the end of the clinical trial [31], which was also related to the scaling procedure, in agreement with the results from the present report.

Molecular characterization of isolates allowed us to establish a bacterial collection of 70 representative *enterococci*. A predominance of *E. faecalis* and *E. faecium* was observed in dental plaque samples, agreeing with other studies on *enterococci* from healthy dogs [8,9,10,35]. In the animals submitted to nisin–biogel treatment (TG), it was possible to observe a reduction of *Enterococcus* spp.; however, animals from both test groups (TG and CG) showed similar species distribution at the end of the clinical trial (Figure 1).

Several virulence determinants have been identified and studied in *enterococci* from different origins [36,37,38,39,40]. The production of virulence factors has been associated with host immune system evasion, degradation of substrates, and adhesion to cell surfaces, favouring disease establishment [41]. In our study, biofilm forming ability was the most prevalent virulent determinant, followed by lipase production and haemolytic activity (Table 3). Several studies have reported similar virulence factors prevalences [42,43]. Biofilm forming ability is linked to an increased antimicrobial resistance profile and recalcitrant conditions, impairing bacterial infections resolution via conventional treatments [44,45]. The complex biofilm environment allows bacteria to interact and exchange genetic material, protects them from the host immune system and from antimicrobials inhibitory action and helps bacterial survival and dissemination [46,47]. Along with a high biofilm ability, the isolates from this study presented mean virulence indexes ranging from 0.39 to 0.49 (Table 3), which may indicate that the *enterococci* under study have the potential to become pathogenic, as stated by Singh at al. [48]. In addition, it was possible to observe an increase in the mean virulence index of the isolates from the animals of the treatment group from T0 to T90, which was not observed in the control group. This may suggest that the nisin–biogel application may have contributed to a slight virulence increase, probably by inducing selective pressure in the oral cavity.

Along with virulence evaluation, to better understand bacterial pathogenic potential it is essential to study their antimicrobial resistance signatures. Antimicrobial resistance (AMR) is a major health problem of our time. Before applying new antimicrobial compounds, as the case of the nisin-biogel, it is essential to study their potential contribution to AMR development. A previous in vitro study has proposed that nisin may lead to an increase in antimicrobial resistance in oral *enterocicci* [23]. In this study, a MDR prevalence of 90 to 100% was detected among the isolates, independently of the timepoint or test group of the associated samples. Previous studies also have described high resistance profiles in canine *enterococci*, with Bertelloni et al. [9] observing an MDR prevalence of 61% and Stepien-Pysniak et al. [10] describing a MDR prevalence of 86% in *enterococci* obtained from healthy dogs. In addition, Cunha et al. (2020) [21] observed that 75% of the *enterococci* obtained from the oral cavity of dogs with PD had an MDR profile. These results reinforce the relevance of *enterococci* in AMR dissemination and maintenance.

Considering the AMR topic, the high-level aminoglycoside resistance of the *enterococci* collection was also evaluated, using gentamycin and streptomycin. It is known that *enterococci* are intrinsically resistant to aminoglycosides [49]; however, the aminoglycosides gentamycin and streptomycin may be successfully used in combination with β-lactams for the treatment of enterococcal infections if HLAR is not detected [49,50]. In agreement with the MDR profile detected in our study, we observed a parallel increase in HLAR in isolates from both test groups during the clinical trial. Several studies have revealed HLAR prevalences ranging from 21% to 47.1% in *enterococci* from an animal origin, with isolates showing higher resistance to streptomycin [9,10]. In our study, we observed a higher HLAR, but without a significant difference between gentamycin or streptomycin resistance. Sienko and collaborators [46] also reported high HLAR prevalences in human *enterococci* isolates from different countries. Unfortunately, the HLAR is now extremely widespread, and the synergistic effect between β-lactams and aminoglycosides is being lost, hindering the treatment of life-threatening *enterococci* infections [46].

By evaluating the MAR index of the isolates under study, high values were observed in *enterococci* from both groups, with a similar increase during the 90-days trial. The isolates from the control group showed the highest MAR value, being statistically different from the MAR index of the isolates from the TG at T90. According to Singh and collaborators [48], isolates with MAR index ≥0.3 and virulence index ≥0.5 are highly threatening isolates, with a significant pathogenic potential for humans or animals. Considering that, the isolates from our collection may have the potential to became pathogenic. Comparing the results from the control and treatment groups, nisin seemed to have a low influence on in vivo AMR dissemination, which is a promising result supporting its potential clinical application.

This study presented some limitations, including the number of animals that participated in the trial, which could be higher to allow to evaluate a larger number of samples. Despite that, it was possible to isolate *enterococci* from the oral cavity of the majority of the animals under trial, allowing the construction of a relevant bacterial collection.

It is important to notice that, to the authors knowledge, this was the first in vivo evaluation of the potential effect of nisin–biogel on the dissemination of antimicrobial resistance and virulence bacterial patterns. When compared with in vitro studies, a low effect on *Enterococcus* antimicrobial and virulence signatures was observed, which reinforces that nisin–biogel is a valuable compound to be used for PD control.

## 4. Materials and Methods

### 4.1. Nisin–Biogel Preparation

Nisin–biogel was prepared as described elsewhere [3,19,20,26,51]. Briefly, a nisin solution of 1000 μg/mL was obtained by dissolving 1 g of nisin powder (2.5% purity, 1000 IU/mg, Sigma-Aldrich, St. Louis, MO, USA) in 25 mL of HCl (0.02 M) (Merck, Alges, Portugal). A 1.5% guar-gum biogel (*w*/*v*) solution was obtained by dissolving 0.75 g of guar gum (Sigma-Aldrich, St. Louis, MO, USA) in 50 mL of sterile distilled water, which was then heat sterilized via an autoclave. Afterwards, nisin was incorporated within the biogel in a proportion of 1:1, in order to obtain a gel with a final concentration of 200 µg/mL to be used in the clinical trial [26,31]. The nisin–biogel concentration was selected based on previous reports regarding its cytotoxicity [20]. For posology establishment, standard procedures for PD prevention were considered [52,53].

### 4.2. Clinical Trial and Sample Collection

**Figure 2:**
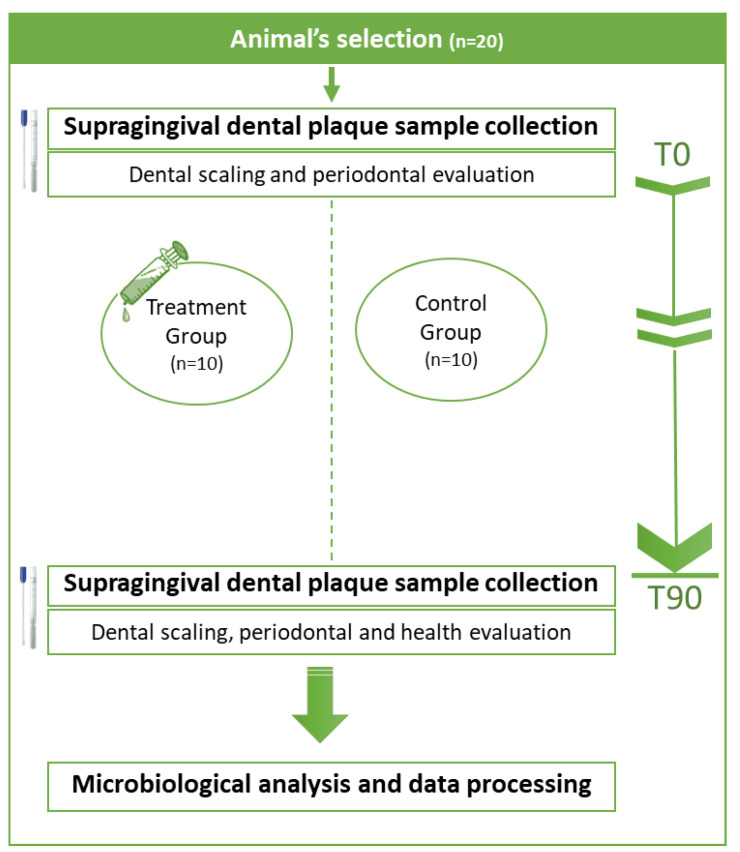
Schematic representation of the clinical trial.

All samples used in this study were obtained from a clinical trial previously performed by our team (Figure 2) [48]. In this former trial, a total of 20 dogs were selected from an official animal’s rescue institution, according to the Veterinary Oral Health Council (VOHC) instructions for testing compounds aiming at PD prevention. The clinical trial was approved by the Ethical Committee for Research and Teaching (CEIE) of the Faculty of Veterinary Medicine, University of Lisbon, Portugal (N/Ref 014/2020) [31].

Briefly, the dogs that participated in this study were healthy dogs, more than 2 years old, without severe PD and no history of antimicrobial therapy in the previous month. All dogs were housed in the same facilities and had access to the same food and housing routines. Prior to the study, all animals were submitted to a clinical examination and oral handling/cleaning, and a complete blood analysis to detect any deviations that would impair their inclusion in the study was performed. Then, the animals were randomly allocated to one of two groups: the treatment group (*n* = 10) or control group (*n* = 10). Animals from the treatment group were submitted to the dental topical application of the nisin–biogel (200 µg/mL) every 48 h; animals from the control group were not submitted to any treatment. Animals were kept in this trial for 90 days [31].

All dogs were submitted to a supragingival dental plaque sample collection using a swab (AMIES^®^, VWR, Amadora, Portugal), applied to all of the dental surface, at day 0 (Timepoint 0—T0) and at day 90 (Timepoint 90—T90). Swabs were transported to the Laboratory of Microbiology and Immunology, Faculty of Veterinary Medicine, University of Lisbon, and processed for *Enterococcus* spp. isolation, quantification, identification, and characterization, according to Semedo-Lemsaddek et al. [37], Oliveira et al. [8] and Belo et al. [54].

### 4.3. Enterococci Identification and Typing

Slanetz and Bartley agar medium (SBA, PanReac AppliChem, Barcelona, Spain) was used for performing a presumptive selection and quantification of *enterococci*, by using ten-fold serial dilutions, a medium inoculation and determination of the colony forming units [8,37]. From each animal and timepoint (T0 and T90), four typical single colonies presenting distinct morphologies were randomly selected from the SBA plates for further characterization using conventional microbiological procedures [8]. Then, molecular identification at the genus level was performed via conventional PCR, according to Ke et al. [55]. Identification at species level was performed via a multiplex PCR using species-specific primers and conditions previously described by Jackson et al. [51]. Genomic typing was performed using the primers OPC19 and (GTG)_5_ in independent mixtures, as described by Semedo-Lemsaddek et al. [37] and Oliveira et al. [8]. The profiles obtained were analysed using BioNumerics^®^ 6.6 (Applied Maths, Kortrijk, Belgium), through a hierarchical numerical approach with Pearson correlation coefficient (optimization 1.5%) and the unweighted pair group method with arithmetic average (UPGMA) as the agglomerative clustering. The isolates’ similarity was evaluated using a composite analysis of the fingerprints obtained with the two primers. The cut level applied to select representative isolates from each timepoint (T0 and T90) was 95.9%, since it was the reproducibility value determined as the average similarity value of all replicate pairs. The selected isolates were further evaluated regarding their virulence and antimicrobial resistance signatures. All primers used in this study are presented in Table 3.

**Table 3:** Description of all primers used for the *enterococci* genus and species identification and genomic typing.

Target	Primers	Sequence	Product Size	Reference
*Enterococcus* spp.	Ent 1	5′-TACTGACAAACCATTCATGATG-3′	112 bp	[50]
	Ent 2	5′-AACTTCGTCACCAACGCGAAC-3′		
*E. faecalis*	FL1	5′-ACTTATGTGACTAACTTAACC-3′	360 bp	[56]
	FL2	5′-TAATGGTGAATCTTGGTTTGG-3′		
*E. faecium*	FM1	5′-GAAAAAACAATAGAAGAATTAT-3′	215 bp	
	FM2	5′-TGCTTTTTTGAATTCTTCTTTA-3′		
*E. hirae*	HI1	5′-CTTTCTGATATGGATGCTGTC-3′	187 bp	
	HI2	5′-TAAATTCTTCCTTAAATGTTG-3′		
*E. durans*	DU1	5′-CCTACTGATATTAAGACAGCG-3′	295 bp	
	DU2	5′-TAATCCTAAGATAGGTGTTTG-3′		
Genotyping	OPC	5′-GTTGCCAGCC-3′	[200–3000]	[8,37]
	(GTG)5	5‘-GTGGTGGTGGTGGTG-3′	[200–3000]	

### 4.4. Enterococci Virulence Signature

The production of gelatinase, lipase, DNase, lecithinase, proteinase, hemolysin and biofilm were evaluated using plate assays, according to Freeman et al. [57], Semedo-Lemsaddek et al. [37] and Fernandes et al. [58]. Virulence factors were evaluated after streaking the colonies onto the respective medium and incubating at 37 °C for 48 h, except for haemolysis and biofilm evaluation.

Briefly, for gelatinase activity evaluation, the Nutrient Gelatin Medium (Oxoid, Basingstoke, Hampshire, UK) was used, with the presence of a liquid medium after incubation indicating a positive result. Lipase production was evaluated using Spirit blue agar and lipase reagent (BD Life Sciences, VWR, Amadora, Portugal), with lipolytic organisms showing a white clearing beneath and surrounding the colonies after incubation. The ability to produce DNase was evaluated using DNAse test agar (Remel, Termo Scientific, Lenexa, KS, USA) supplemented with blue toluidine, and the presence of pink colonies after incubation was considered a positive result. Lecitinase production was determined using Tryptic Soy agar (VWR, Leuven, Belgium) supplemented with 10% of egg yolk emulsion (VWR, Leuven, Belgium), and a positive activity resulted in the development of a white precipitate around colonies after incubation. Proteinase activity was determined using a skim milk medium (VWR, Leuven, Belgium), and colonies presenting a white halo after incubation being considered positive. Hemolysin production was evaluated after isolates streaking on Columbia agar supplemented with 5% sheep blood (Biomeriux, Marcy-l’Étoile, France), incubated at 37 °C for 72 h in anaerobic conditions. The presence of a clearing halo around the colonies was interpreted as a positive result (β-haemolysis), and the absence of a clearing (ɣ-haemolysis) or the presence of a greenish zone around the colonies (α-haemolysis) were considered negative results. Biofilm forming ability was evaluated using Congo Red agar medium (Sigma-Aldrich, St. Louis, MO, USA), incubated for 72 h at 37 °C, after which the development of black colonies indicated the isolates’ ability to produce biofilm. According to the time required for biofilm production, isolates were considered as strong (24 h), moderate (48 h) or weak biofilm producers (72 h). The evaluation of the isolate’s phenotypic virulence profile included the determination of their virulence index (number of positive virulence factors/number of virulence factors tested).

### 4.5. Enterococci Antimicrobial Resistance Profile

Isolates’ antimicrobial resistance profile was determined using the disk diffusion method according to the guidelines of the Clinical and Laboratory Standards Institute (CLSI) (M100S and Vet01-02) [59,60]. A total of 12 antimicrobials (Oxoid, Hampshire, UK), presented in Table 4, were used, being selected based on their relevance to veterinary medicine, as well as to public health.

Isolates were categorised according to their multidrug-resistant patterns, as described by Magiorakos et al. [24], and by their multiple antibiotic resistance (MAR) indices (number of antibiotics to which isolates were resistant/number of antibiotics tested) [58,61]. In addition, isolates with non-susceptibility to high doses of gentamicin (120 µg) and/or streptomycin (300 µg) were considered to present high-level aminoglycoside resistance (HLAR) [50,59,60].

### 4.6. Statistical Analysis

Data statistical analysis was carried out using Microsoft Excel 2016^®^. Differences in the virulence and MAR index between groups and timepoints were evaluated using Student’s *t* test.

Quantitative variables are expressed as mean values ± standard deviation. A confidence interval of 95% was considered, with a *p*-value ≤ 0.05 indicating statistical significance.

## 5. Conclusions

*Enterococci* are organisms with an impressive genetic versatility and unparalleled ability to recruit and express antimicrobial resistance and virulence determinants. Their commensal nature allows them to colonize healthy individuals and quickly participate in complicated infections. Present in the oral cavity, they are considered an interesting bacterial model to be used in odontology and pharmaceutical research, since they can act as reservoirs of resistant and virulent phenotypes. The long-term dental application of the nisin–biogel to dogs showed to be an interesting alternative to be used for PD control, with a low effect on *Enterococcus* antimicrobial and virulence profiles.

Considering all the results obtained in this study regarding the dental application of the nisin–biogel to dogs, in the future, this compound could be commercially developed to be used by clinicians in PD management in dogs.

## Figures and Tables

**Table 1:** Number of isolates with positive phenotypic expression of the virulence factors under study, by group and timepoint, and mean virulence index.

Virulence Factor	Control Group		Treatment Group	
	Timepoint 0	Timepoint 90	Timepoint 0	Timepoint 90
Gelatinase	7	4	5	5
Lipase	14	13	9	18
DNase	0	0	0	0
Lecithinase	7	3	4	5
Haemolysin	10	5	15	12
Proteinase	6	3	4	5
Biofilm	18	13	17	19
Total of isolates evaluated	18	13	20	19
Mean virulence index ± SD	0.49 ± 0.24	0.45 ± 0.24	0.39 ± 0.24	0.48 ± 0.24

SD: Standard deviation.

**Table 2:** Antimicrobial resistance evaluation of isolates under study by timepoint and test group.

		Control Group		Treatment Group	
		Timepoint 0	Timepoint 90	Timepoint 0	Timepoint 90
Mean MAR index ± SD		0.62 ± 0.20	0.83 ± 0.15	0.40 ± 0.22	0.61 ± 0.18
Number of isolates with MDR profile		18	13	18	19
Number of isolates with HLAR	CN + S	7	9	2	3
	CN	1	0	1	4
	S	3	2	0	3
Total of isolates evaluated		18	13	20	19

MAR: Multiple antimicrobial resistance; MDR: multidrug resistance; HLAR: high-level aminoglycoside resistance; CN: gentamycin; S: streptomycin; SD: standard deviation.

**Table 4:** Antimicrobial agents used in the *enterococci* antimicrobial susceptibility profiling, grouped by mechanism of action, class and concentration.

Mechanism of Action	Antimicrobial Class	Antimicrobial Drug	Concentration (µg Per Disk)
Inhibition of cell-wall synthesis	Aminopenicillins	Ampicillin (AMP)	10
		Amoxicillin/Clavulanate (AMX)	30
	Glycopeptides	Vancomycin (VA)	30
	Carbapenems	Imipenem (IMI)	10
Inhibition of nucleic acid synthesis	Fluoroquinolones	Enrofloxacin (ENR)	5
Inhibition of protein synthesis	Tetracyclines	Tetracycline (T)	30
		Doxycycline (DTX)	30
	Aminoglycosides	Gentamicin (CN)	120
		Streptomycin (S)	300
	Macrolides	Erythromycin (E)	15
	Lincosamide	Clindamycin (DA)	2
	Nitrobenzenes	Chloramphenicol (C)	30

## Data Availability

The datasets used and/or analyzed in the current study are available from the corresponding author upon reasonable request.

## Supplementary Materials

The following supporting information can be downloaded at: https://www.mdpi.com/article/10.3390/antibiotics12030468/s1, Supplementary File S1: Dendrograms based on the composite analysis of the isolates fingerprints with primers OPC19 and (GTG)5, using Pearson correlation coefficient.
