# Supplementary material for: In Vivo Effect of a Nisin–Biogel on the Antimicrobial and Virulence Signatures of Canine Oral Enterococci
Source: Antibiotics (Basel). 2023 Feb 25;12(3):468. doi: 10.3390/antibiotics12030468 (PMC10044209; doi:10.3390/antibiotics12030468)
Supplement: Supplementary file 1 [file antibiotics-12-00468-s001.zip › antibiotics-2222122-supplementary.pdf]

Supplementary file S1: Dendrograms based on the composite analysis of the isolates fingerprints with primers OPC19 and (GTG)5, using the Pearson correlation coefficient. A) Dendrogram including all isolates from Timepoint 0. B) Dendrogram including all isolates from Timepoint 90. C) Dendrogram including the representative isolates from Timepoint 0. D) Dendrogram including representative isolates from Timepoint 90.

A

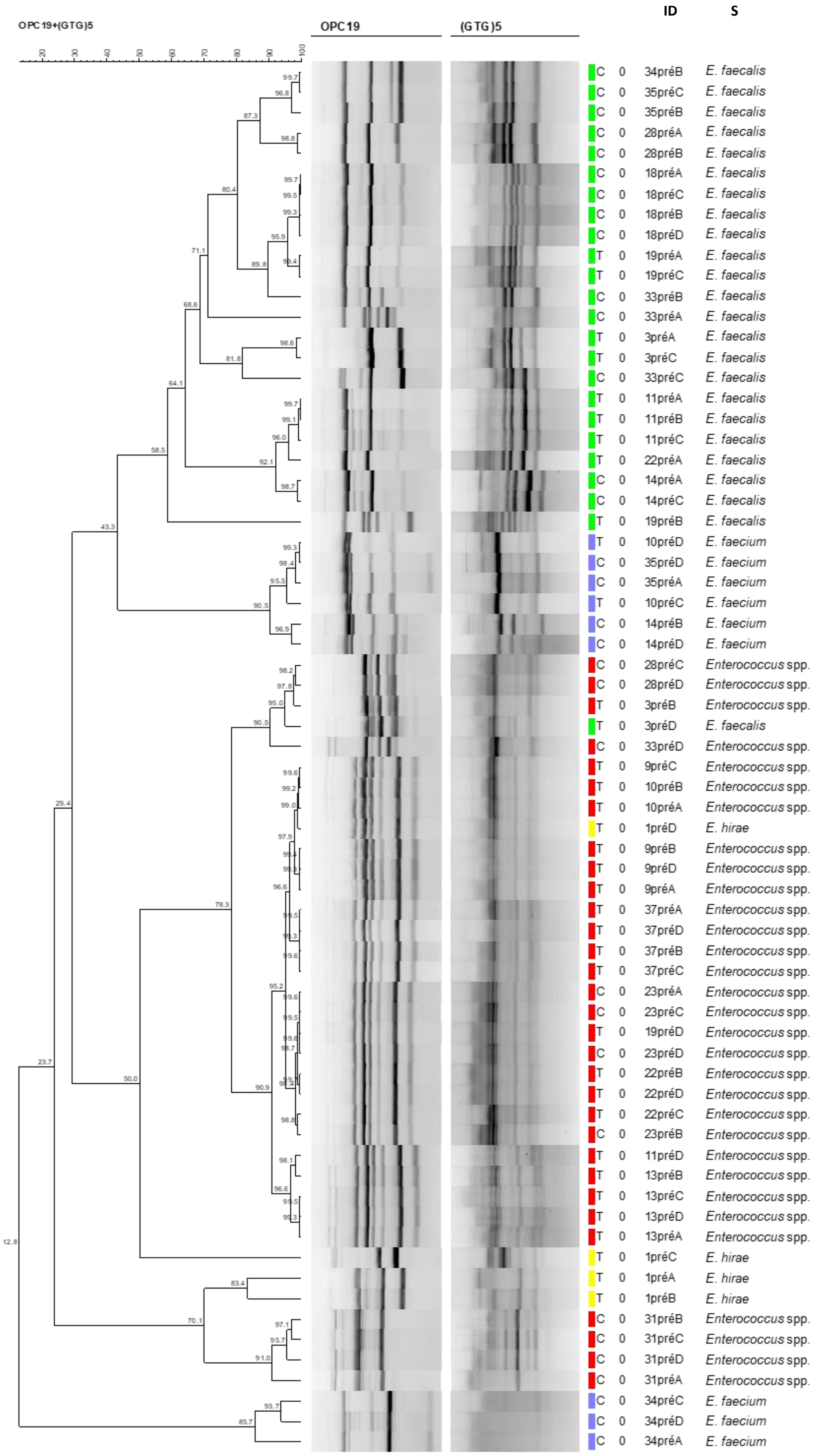

OPC19+(GTG)5
OPC19
(GTG)5
ID
S
C 0 34préB E. faecalis
C 0 35préC E. faecalis
C 0 35préB E. faecalis
C 0 28préA E. faecalis
C 0 28préB E. faecalis
C 0 18préA E. faecalis
C 0 18préC E. faecalis
C 0 18préB E. faecalis
C 0 18préD E. faecalis
T 0 19préA E. faecalis
T 0 19préC E. faecalis
C 0 33préB E. faecalis
C 0 33préA E. faecalis
T 0 3préA E. faecalis
T 0 3préC E. faecalis
C 0 33préC E. faecalis
T 0 11préA E. faecalis
T 0 11préB E. faecalis
T 0 11préC E. faecalis
T 0 22préA E. faecalis
C 0 14préA E. faecalis
C 0 14préC E. faecalis
T 0 19préB E. faecalis
T 0 10préD E. faecium
C 0 35préD E. faecium
C 0 35préA E. faecium
T 0 10préC E. faecium
C 0 14préB E. faecium
C 0 14préD E. faecium
C 0 28préC Enterococcus spp.
C 0 28préD Enterococcus spp.
T 0 3préB Enterococcus spp.
T 0 3préD E. faecalis
C 0 33préD Enterococcus spp.
T 0 9préC Enterococcus spp.
T 0 10préB Enterococcus spp.
T 0 10préA Enterococcus spp.
T 0 1préD E. hirae
T 0 9préB Enterococcus spp.
T 0 9préD Enterococcus spp.
T 0 9préA Enterococcus spp.
T 0 37préA Enterococcus spp.
T 0 37préD Enterococcus spp.
T 0 37préB Enterococcus spp.
T 0 37préC Enterococcus spp.
C 0 23préA Enterococcus spp.
C 0 23préC Enterococcus spp.
T 0 19préD Enterococcus spp.
C 0 23préD Enterococcus spp.
T 0 22préB Enterococcus spp.
T 0 22préD Enterococcus spp.
T 0 22préC Enterococcus spp.
C 0 23préB Enterococcus spp.
T 0 11préD Enterococcus spp.
T 0 13préB Enterococcus spp.
T 0 13préC Enterococcus spp.
T 0 13préD Enterococcus spp.
T 0 13préA Enterococcus spp.
T 0 1préC E. hirae
T 0 1préA E. hirae
T 0 1préB E. hirae
C 0 31préB Enterococcus spp.
C 0 31préC Enterococcus spp.
C 0 31préD Enterococcus spp.
C 0 31préA Enterococcus spp.
C 0 34préC E. faecium
C 0 34préD E. faecium
C 0 34préA E. faecium

B

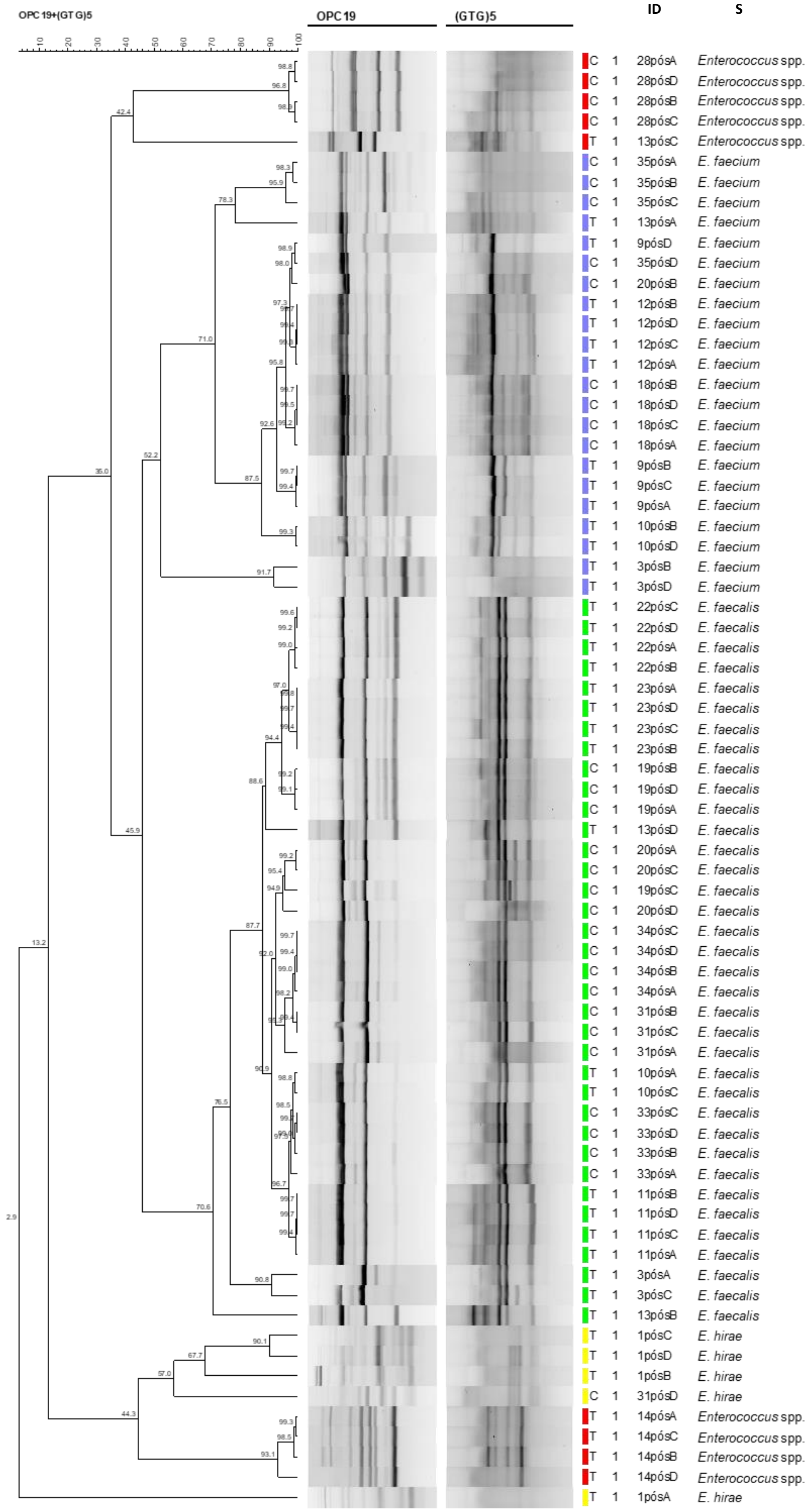
OPC 19+(GTG)5
OPC19
(GTG)5
ID
S
C 1 28pósA Enterococcus spp.
C 1 28pósD Enterococcus spp.
C 1 28pósB Enterococcus spp.
C 1 28pósC Enterococcus spp.
T 1 13pósC Enterococcus spp.
C 1 35pósA E. faecium
C 1 35pósB E. faecium
C 1 35pósC E. faecium
T 1 13pósA E. faecium
T 1 9pósD E. faecium
C 1 35pósD E. faecium
C 1 20pósB E. faecium
T 1 12pósB E. faecium
T 1 12pósD E. faecium
T 1 12pósC E. faecium
T 1 12pósA E. faecium
C 1 18pósB E. faecium
C 1 18pósD E. faecium
C 1 18pósC E. faecium
C 1 18pósA E. faecium
T 1 9pósB E. faecium
T 1 9pósC E. faecium
T 1 9pósA E. faecium
T 1 10pósB E. faecium
T 1 10pósD E. faecium
T 1 3pósB E. faecium
T 1 3pósD E. faecium
T 1 22pósC E. faecalis
T 1 22pósD E. faecalis
T 1 22pósA E. faecalis
T 1 22pósB E. faecalis
T 1 23pósA E. faecalis
T 1 23pósD E. faecalis
T 1 23pósC E. faecalis
T 1 23pósB E. faecalis
C 1 19pósB E. faecalis
C 1 19pósD E. faecalis
C 1 19pósA E. faecalis
T 1 13pósD E. faecalis
C 1 20pósA E. faecalis
C 1 20pósC E. faecalis
C 1 19pósC E. faecalis
C 1 20pósD E. faecalis
C 1 34pósC E. faecalis
C 1 34pósD E. faecalis
C 1 34pósB E. faecalis
C 1 34pósA E. faecalis
C 1 31pósB E. faecalis
C 1 31pósC E. faecalis
C 1 31pósA E. faecalis
T 1 10pósA E. faecalis
T 1 10pósC E. faecalis
C 1 33pósC E. faecalis
C 1 33pósD E. faecalis
C 1 33pósB E. faecalis
C 1 33pósA E. faecalis
T 1 11pósB E. faecalis
T 1 11pósD E. faecalis
T 1 11pósC E. faecalis
T 1 11pósA E. faecalis
T 1 3pósA E. faecalis
T 1 3pósC E. faecalis
T 1 13pósB E. faecalis
T 1 1pósC E. hirae
T 1 1pósD E. hirae
T 1 1pósB E. hirae
C 1 31pósD E. hirae
T 1 14pósA Enterococcus spp.
T 1 14pósC Enterococcus spp.
T 1 14pósB Enterococcus spp.
T 1 14pósD Enterococcus spp.
T 1 1pósA E. hirae

C

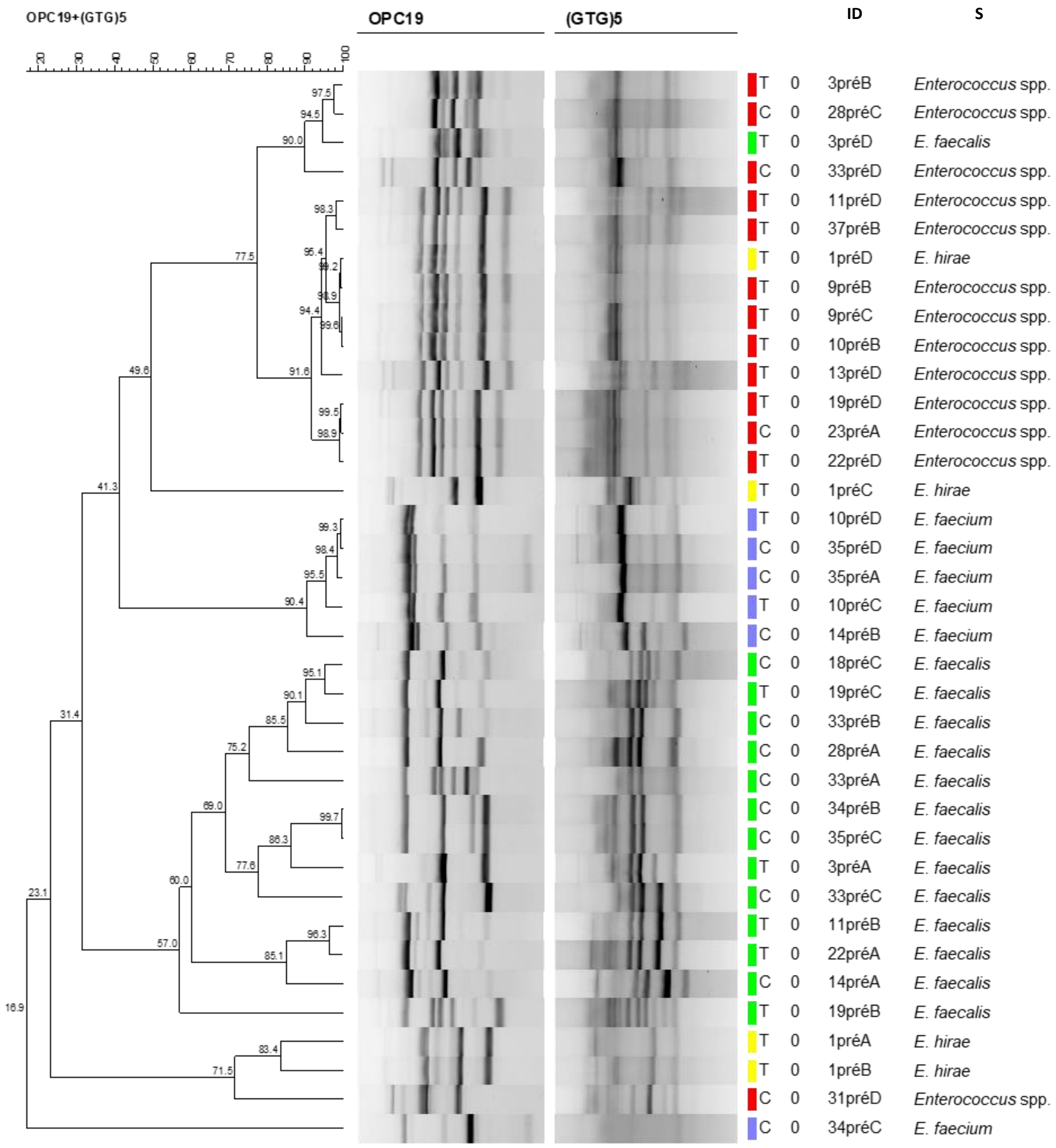

OPC19+(GTG)5
OPC19
(GTG)5
ID
S
T 0 3préB Enterococcus spp.
C 0 28préC Enterococcus spp.
T 0 3préD E. faecalis
C 0 33préD Enterococcus spp.
T 0 11préD Enterococcus spp.
T 0 37préB Enterococcus spp.
T 0 1préD E. hirae
T 0 9préB Enterococcus spp.
T 0 9préC Enterococcus spp.
T 0 10préB Enterococcus spp.
T 0 13préD Enterococcus spp.
T 0 19préD Enterococcus spp.
C 0 23préA Enterococcus spp.
T 0 22préD Enterococcus spp.
T 0 1préC E. hirae
T 0 10préD E. faecium
C 0 35préD E. faecium
C 0 35préA E. faecium
T 0 10préC E. faecium
C 0 14préB E. faecium
C 0 18préC E. faecalis
T 0 19préC E. faecalis
C 0 33préB E. faecalis
C 0 28préA E. faecalis
C 0 33préA E. faecalis
C 0 34préB E. faecalis
C 0 35préC E. faecalis
T 0 3préA E. faecalis
C 0 33préC E. faecalis
T 0 11préB E. faecalis
T 0 22préA E. faecalis
C 0 14préA E. faecalis
T 0 19préB E. faecalis
T 0 1préA E. hirae
T 0 1préB E. hirae
C 0 31préD Enterococcus spp.
C 0 34préC E. faecium

D

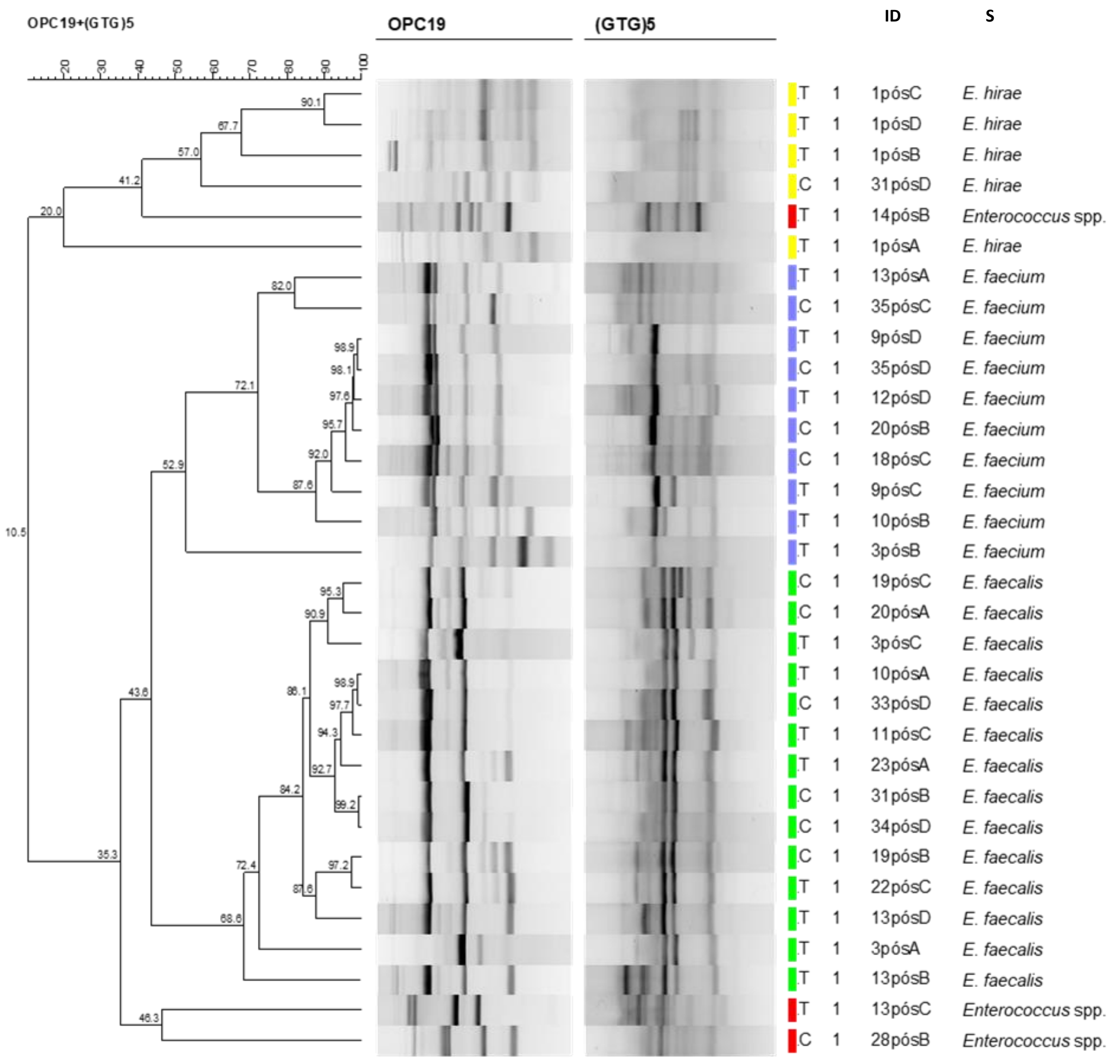

Legend: T- treatment group; C- Control Group; 0 – Timepoint 0; 1- Timepoint 90; ID – Animal identification; S – Species identification.
